# Coxsackievirus B3 Infection of Human Neural Progenitor Cells Results in Distinct Expression Patterns of Innate Immune Genes

**DOI:** 10.3390/v12030325

**Published:** 2020-03-17

**Authors:** Soo-Jin Oh, Jeong-An Gim, Jae Kyung Lee, Hosun Park, Ok Sarah Shin

**Affiliations:** 1 Department of Biomedical Sciences, BK21 PLUS program, College of Medicine, Korea University Guro Hospital, Seoul 08308, Korea; sjooooh@gmail.com (S.-J.O.); jae.lee0321@gmail.com (J.K.L.); 2 Medical Science Research Center, College of Medicine, Korea University Guro Hospital, Seoul 08308, Korea; vitastar@korea.ac.kr; 3 Department of Microbiology, College of Medicine, Yeungnam University, 170 Hyeonchung-ro, Namgu, Daegu 42415, Korea

**Keywords:** Coxsackievirus B3 (CVB3), gene expression profiles, neuronal progenitor cells, interferons, suppressor of cytokine signaling

## Abstract

Coxsackievirus B3 (CVB3), a member of *Picornaviridae* family, is an important human pathogen that causes a wide range of diseases, including myocarditis, pancreatitis, and meningitis. Although CVB3 has been well demonstrated to target murine neural progenitor cells (NPCs), gene expression profiles of CVB3-infected human NPCs (hNPCs) has not been fully explored. To characterize the molecular signatures and complexity of CVB3-mediated host cellular responses in hNPCs, we performed QuantSeq 3′ mRNA sequencing. Increased expression levels of viral RNA sensors (*RIG-I*, *MDA5*) and interferon-stimulated genes, such as *IFN-β*, *IP-10*, *ISG15*, *OAS1*, *OAS2*, *Mx2*, were detected in response to CVB3 infection, while *IFN-γ* expression level was significantly downregulated in hNPCs. Consistent with the gene expression profile, CVB3 infection led to enhanced secretion of inflammatory cytokines and chemokines, such as interleukin-6 (IL-6), interleukin-8 (IL-8), and monocyte chemoattractant protein-1 (MCP-1). Furthermore, we show that type I interferon (IFN) treatment in hNPCs leads to significant attenuation of CVB3 RNA copy numbers, whereas, type II IFN (IFN-γ) treatment enhances CVB3 replication and upregulates suppressor of cytokine signaling 1/3 (SOCS) expression levels. Taken together, our results demonstrate the distinct molecular patterns of cellular responses to CVB3 infection in hNPCs and the pro-viral function of IFN-γ via the modulation of SOCS expression.

## 1. Introduction

Coxsackievirus B3 (CVB3) belongs to *Enterovirus* genus within the *Picornaviridae* family of viruses, associated with a wide variety of diseases, ranging from mild flu like symptoms to severe diseases including myocarditis, pancreatitis, and type I diabetes [1]. CVB3 can cause severe morbidity and mortality, particularly in younger patients, and infection during pregnancy can result in susceptibility to spontaneous abortion, fetal myocarditis, and neurodevelopmental defects in neonates [2,3]. In addition, the neonatal central nervous system (CNS) and heart are major targets of CVB3 infection. Previously, Feuer and colleagues have shown, using animal models, that CVB3 preferentially targets neural progenitor cells (NPCs) in the CNS [4,5,6,7,8]. In particular, they suggested that NPCs and neurogenic regions of the CNS may support persistent CVB3 infection and infected mice surviving infection may suffer a chronic depletion of neural stem cells. The effect of CVB3 infection in human neural progenitor cell (hNPC) model, however, remains to be investigated.

CVB3 has evolved many unique mechanisms to evade the innate immune response [1]. Nonetheless, the induction of type I interferon (IFN) signaling is essential for the control of CVB3 infection, as evident from the enhanced virus-induced lethality in type I IFN receptor null mice [9] and the increased susceptibility to CVB3 infection in IFN-β-deficient mice [10]. Both melanoma differentiation-associated gene 5 (MDA5)-and mitochondria antiviral-signaling protein (MAVS)-mediated type I IFN signaling pathways have been implicated in the response to CVB3 infections, and mice deficient in either TIR-domain-containing adapter-inducing interferon-β (TRIF) or MAVS show an enhanced susceptibility to viral infection [11]. CVB3-induced innate immune responses in human neural progenitor cells (hNPCs), however, are currently unknown.

Here, we have characterized CVB3-induced cellular responses in hNPCs, providing a comprehensive measurement of the host gene expression in a post-infection time-dependent manner. Furthermore, our data demonstrate that type I IFN treatment contributes to the attenuation of CVB3 replication, whereas type II IFN enhances CVB3 replication via the upregulation of suppressor of cytokine signaling (SOCS1/3) expression.

## 2. Materials and Methods

### 2.1. Cells and Viruses

HeLa cells purchased from American Type Culture Collection (Manassas, VA, USA) were maintained in Dulbecco’s modified Eagle’s medium (DMEM; Corning Mediatech, Corning, NY, USA) supplemented with 10% fetal bovine serum (FBS; Corning) and antibiotics.

The preparation of hNPCs was as described previously [12,13]. Briefly, human embryonic stem cells (hESCs) were grown under standard culture conditions with a feeder layer and transferred to a matrigel-coated plate with mouse embryonic fibroblasts (MEFs) conditioned medium. After two consecutive passages, neural differentiation was induced by replacing the hESC medium with DMEM/F12 supplemented with 2% B27, 100 ng/mL fibroblast growth factor, 100 ng/mL epidermal growth factor and 5 μg/mL heparin. Partially differentiated hESCs were dissociated with accutase and plated on geltrex-coated plates. Homogenous populations of NPCs were obtained after three continuous passages. Matrigel was purchased from BD Biosciences (San Jose, CA, USA) and other reagents were purchased from Invitrogen (Carlsbad, CA, USA).

CVB3 (H3 strain, Woodruff variant) [14] was a generous gift of Jae-Hwan Nam (Catholic University of Korea) and quantified by plaque assay on HeLa cells, as previously described [15].

### 2.2. Cell Viability Assay

Cells were seeded in 96-well plates. After 24 h, media were changed and CVB3 was infected for various time points. Cell viability was determined using CellTiter-Glo 2.0 luminescence assay (Promega, Madison, WI, USA) following manufacturer’s recommendations.

### 2.3. Gene Expression Analysis Using QuantSeq 3′ mRNA Sequencing

Total RNA was isolated using Trizol reagent (Invitrogen), as previously reported [13,16]. RNA quality was assessed by Agilent 2100 bioanalyzer using RNA 6000 Nano Chip (Agilent Technologies, Amstelveen, The Netherlands), and RNA quantification was performed using ND-2000 Spectrophotometer (Thermo Fisher Scientific, Waltham, MA, USA). The construction of library was performed using QuantSeq 3′ mRNA-Seq Library Prep Kit (Lexogen, Inc., Vienna, Austria) according to manufacturer’s instructions. In brief, each 500 ng total RNA was prepared for hybridization with the oligo-dT primer containing an Illumina-compatible sequence at the 5′ end. Reverse transcription was performed. After degradation of the RNA template, a second strand synthesis was initiated by a random primer containing an Illumina-compatible linker sequence at the 5′ end. All reaction components were removed using magnetic beads for library purification. Amplification of the library added complete adapter sequences required for cluster generation. The finished library was purified from PCR components and high-throughput sequencing was performed as single-end 75 bp sequencing using NextSeq 500 (Illumina, Inc., San Diego, CA, USA). QuantSeq 3′ mRNA reads were aligned using Bowtie2 [17]. Bowtie2 indices were generated either from the genome assembly sequence, or the representative transcript sequences for alignment to the genome and transcriptome. The alignment file was used to assemble and estimate the abundance of transcripts, and detect the differential expression of genes. Differentially expressed genes (DEGs) were determined based on counts from unique and multiple alignments using coverage in Bedtools [18]. The read count data were processed based on the quantile normalization method using EdgeR [19]. Heatmaps were generated and clustering was performed using custom R scripts. Gene Ontology (GO) and KEGG pathway enrichment analyses were performed using DAVID [20] and PANTHER [21]. Functional GO and network analysis of DEG was performed on plugin ClueGO (2.5) [22] and visualized using the plugin *CluePedia (1.5)* embedded in Cytoscape (3.6.0) [23]. Principal Component Analysis was performed using custom R scripts. The raw QuantSeq data files were deposited in NCBI’s Gene Expression Omnibus (GEO) and are accessible via the GEO Series accession number GSE136734.

### 2.4. Real-Time Reverse Transcriptase-Polymerase Chain Reaction (RT-PCR) Assay

QuantSeq results were validated by RT-PCR on specific target genes. First-strand cDNA was synthesized from 0.5 μg of total RNA using ImProm-II Reverse Transcription System (Promega) according to manufacturer’s instructions with the previously described primer sequences [13,24]. QuantStudio 6 Flex Real-time PCR system (Thermo Fisher Scientific) was utilized for cDNA amplification with Power SYBR^®^ Green Master Mix (Applied Biosystems) under the following conditions: 95 °C for 10 min, followed by 40 cycles of 95 °C for 30 s and 60 °C for 1 min. Relative mRNA levels were determined using the comparative C_t_ method and normalized against β-actin mRNA.

### 2.5. Plasmid Transfection

HeLa cells were seeded in 12 well plates. The next day, suppressor of cytokine signaling (SOCS)1 or SOCS3 encoding DNA plasmid (Sino biological, Beijing, China) was mixed with polyethylenimine (PEI, Sigma-Aldrich, St. Louis, MO, USA) in Opti-MEM (Thermo Fisher Scientific), and the mixture was incubated for 20 min at room temperature. DNA–PEI mix was then added to the cells.

### 2.6. Confocal Microscopy

Cells were seeded onto coverslips in 24-well plates and transfected with various plasmids, followed by CVB3 infection at various time points. Cells were washed with PBS, fixed with 4% paraformaldehyde (PFA), and permeabilized with 0.1% Triton X-100, as described previously [12,13]. Cells were then stained with anti-Coxsackievirus B3 antibody (1:1000 dilution; Merck, Kenilworth, NJ, USA), followed by anti-mouse Alexa 594 conjugated antibody (Invitrogen). Coverslips were mounted on glass slides using mounting media containing 4,6-diamidino-2-phenylindole (DAPI) and examined using a confocal microscope (LSM700; Carl Zeiss, Oberkochen, Germany).

### 2.7. Western Blot Analysis

Cells were lysed with RIPA buffer (Sigma-Aldrich) supplemented with a protease and phosphatase inhibitor cocktail (Roche, Basel, Switzerland) at the specified time points. Lysate proteins were resolved by sodium dodecyl sulfate-polyacrylamide gel electrophoresis (SDS-PAGE) on 10–12% acrylamide gels. Proteins were transferred onto polyvinylidene difluoride membranes and blocked with 5% (*w*/*v*) skim milk in Tris-buffered saline (0.2 M Tris, 1.36 M NaCl) supplemented with 0.1% (*v*/*v*) Tween-20 (TBS-Tw) for 1 h at room temperature. This was followed by overnight incubation with primary antibodies (Cell Signaling Technology, Danvers, MA, USA) at 4 °C. As a loading control, tubulin or β-actin (Abgent, San Diego, CA, USA) antibody was used. After three washes in TBS-Tw, the membranes were incubated with horseradish peroxidase-conjugated anti-rabbit or anti-mouse IgG secondary antibodies for 1 h at 25 °C. Membranes were washed with TBS-Tw and incubated in Western Lumi Pico solution (ECL solution kit; DoGen, Seoul, Korea). Signals were determined using a Fusion Solo Imaging System (Vilber Lourmat, Collégien, France). Band intensities were quantified by Fusion-Capt analysis software (Vilber Lourmat).

### 2.8. Cytokine Secretion Measurements by ELISA

Cell-free cultured supernatant was examined for interleukin-6 (IL-6), interleukin-8 (IL-8), monocyte chemoattractant protein-1 (MCP-1) concentrations using ELISA kits (R&D Systems, Minneapolis, MN, USA) following manufacturer’s instructions.

### 2.9. Statistical Analysis

The statistical comparisons between the different treatments were performed using an unpaired two-tailed Student’s *t* test or a Mann Whitney test (Graphpad Software, La Jolla, CA, USA) and *p* < 0.05 was considered statistically significant.

## 3. Results

### 3.1. Human Neural Progenitor Cells Are Susceptible to CVB3 Infection

**Figure 1:**
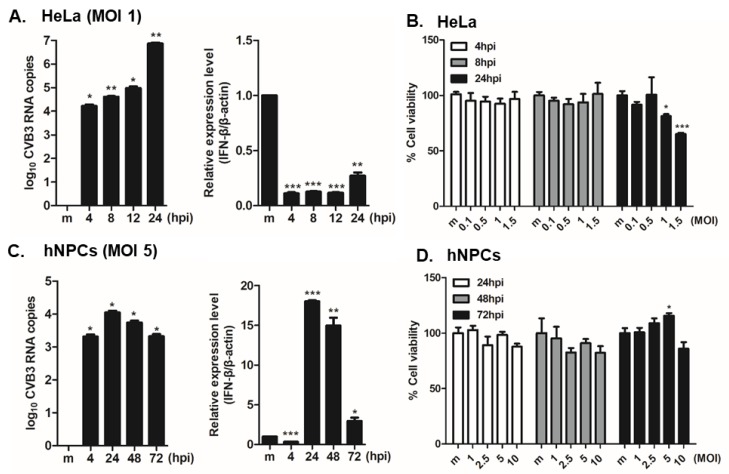
Coxsackievirus B3 (CVB3) efficiently replicates in human neural progenitor cells (hNPCs) and causes minimal cytotoxicity. HeLa cells and hNPCs were infected with CVB3 at MOI of 1 or 5 for the indicated times. (**A**,**C**) RT-PCR was performed to measure CVB3 copy number and *IFN-β* mRNA levels. The expression of viral RNA copies was calculated in relation to the expression level of *β-actin* mRNA. *, *p* < 0.05; **, *p* < 0.01; ***, *p* < 0.001, compared with mock-infected cells. (**B**,**D**) At the end of the incubation period, cell viability was determined using CellTiter Glo assays. Each value represents the mean ± SEM (*n* = 4).

Previous reports have demonstrated that CVB3 preferentially infects murine NPCs in vitro, and that undifferentiated NPCs were more susceptible to CVB3 infection compared to differentiated NPC precursors [4,5,7,8]. Here, we examined the infection patterns of CVB3 in HeLa vs. hNPCs. HeLa cells were infected with CVB3 at an MOI of 1 for specific time points. RT-PCR analysis showed a significant increase of CVB3 RNA copy number in a time-dependent manner in HeLa cells, in comparison to the uninfected control (Figure 1A). On the other hand, *IFN-β* mRNA expression was robustly suppressed upon CVB3 infection in HeLa cells, a finding consistent with a previous report [25]. Furthermore, cell viability results suggest that significant cell death occurs following CVB3 infection at 24 h post infection (hpi) in HeLa cells (Figure 1B). Interestingly, in hNPCs, CVB3 RNA copy numbers increased significantly in a time-dependent manner but HeLa cells were exhibiting higher levels of CVB3 RNA copy number increase, even at lower MOIs. *IFN-β* expression, however, was upregulated only in hNPCs in contrast to HeLa cells (Figure 1C). Quantitative comparison of CVB3-induced cytopathic effect in hNPCs was carried out using a luminescence-based cellular viability assay. Results showed that CVB3-infected hNPCs remain viable at 72 hpi, even at high MOIs (Figure 1D).

### 3.2. Gene Expression Profiling of CVB3-Infected hNPCs

**Figure 2:**
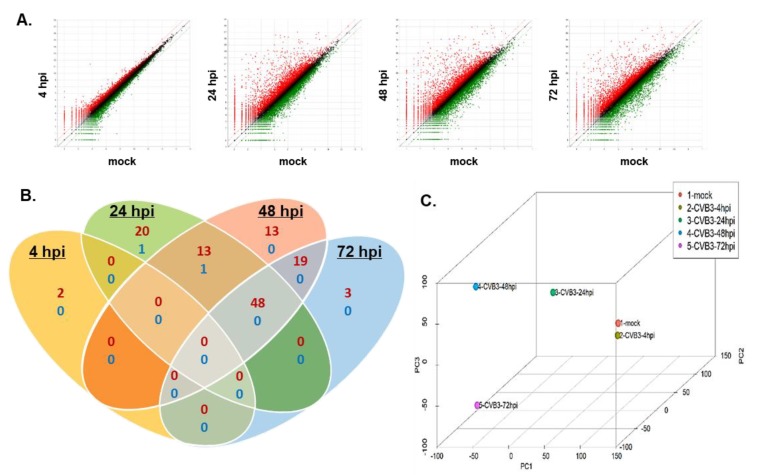
QuantSeq analysis reveals differential expression patterns of host genes in CVB3-infected hNPCs. hNPCs were infected with CVB3 (MOI 5) and RNA was harvested at 4, 24, 48, and 72 h post infection (hpi). (**A**) Scatter plot shows the x-axis and y-axis indicating the expression levels of genes from mock-infected and virus-infected groups, respectively. Relatively highly expressed genes in the virus-infected groups (red) and mock-infected groups (green) are depicted. Black dots represent genes that were not classified as differentially expressed. The differentially expressed gene (DEG) count was identified by comparing mock- and virus-infected groups (fold change > 2) (**B**) Venn diagrams representing the overlap in DEG profiles of CVB3-infected cells. Numbers of upregulated genes (more than 100 folds) are indicated in red and downregulated genes are in blue. (**C**) Three-dimensional multi-dimensional scaling (3D-MDS) plot of QuantSeq datasets shows that 24, 48, and 72 hpi samples are clustered in locations different from the mock or 4 hpi samples.

Alongside microarrays and conventional RNA sequencing, QuantSeq technology provides an additional platform to measure gene expression. To gain insights into the gene expression profiles of CVB3-infected hNPCs, QuantSeq 3′ mRNA sequencing was performed to analyze the transcriptome of CVB3-infected hNPCs at different timepoints. DEGs were identified as genes up- or downregulated with a fold change of ±2 and a *p*-value < 0.05, compared to the mock control. As summarized by expression plots and Venn diagrams, many commonly upregulated DEGs were detected at 24, 48, and 72 hpi, while very few were detected at 4 hpi (Figure 2A,B). To evaluate the degree of dissimilarity among samples in terms of biological variations and dimensions, we performed three dimensional multidimensional scaling (3D-MDS) analysis (Figure 2C). Overall, 24, 48, and 72 hpi samples were distinctly distributed, while 4 hpi and mock samples were relatively close in proximity, as shown in the 3D-MDS plot. These findings indicate that the gene expression profiles of the mock and 4 hpi samples share a degree of similarity, whereas 24, 48, and 72 hpi samples were distinctly clustered.

**Figure 3:**
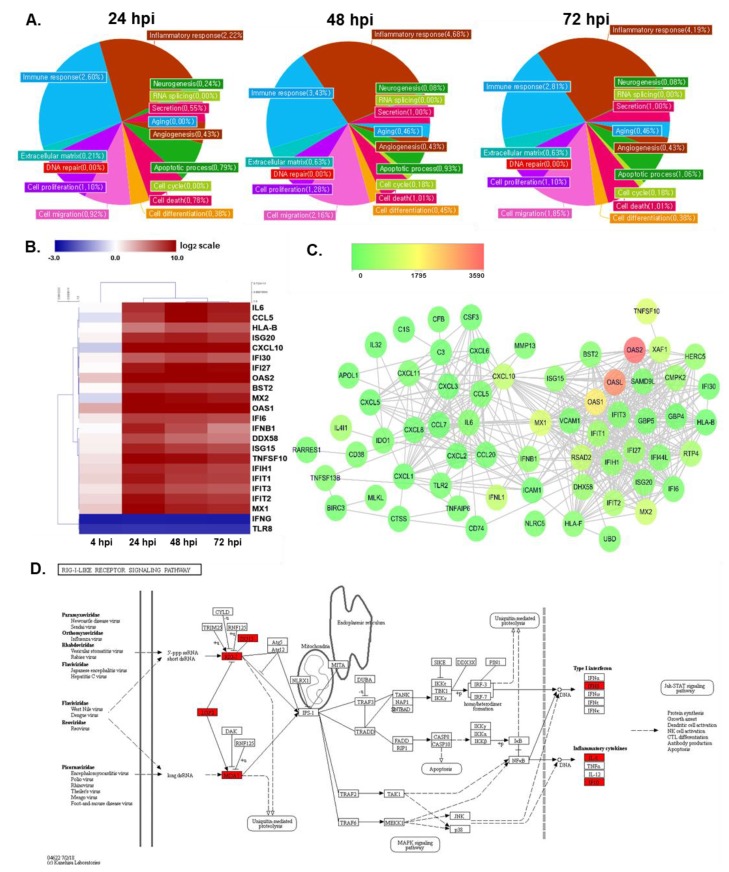
Comprehensive analysis of gene expression profiles of CVB3-infected hNPCs reveals changes in innate immune response genes. (**A**) Gene ontology (**B**) Heatmap indicating the log_2_ fold change values of differentially expressed genes (DEGs) involved in immune responses in response to CVB3 infection of hNPCs, as determined by QuantSeq analysis. Hierarchical clustering was performed to group genes based on similar expression profiles, as indicated by the dendrograms (left). Up- and downregulated DEGs are indicated in red and blue, respectively. Time points are indicated below the panels. (**C**) Interferon-stimulated genes–protein interaction networks of DEGs in 48 hpi samples were constructed using Cytoscape. Nodes represent proteins and edges represent interactions between two proteins. Fold changes in the gene expression of the virus-infected groups was overlaid onto the nodes, as indicated by the color key. (**D**) GG analysis of 48 hpi sample resulted in a list of DEGs involved in RIG-I like receptor signaling pathways. Red, green, and white colors indicate significantly increased, significantly decreased, and unchanged gene expression in mock vs. CVB3-infected cells. DEGs were labelled on the map of RIG-I like receptor signaling pathways obtained from KEGG database with official permission and guidance [26,27,28].

In addition, gene ontology-based bioinformatics analysis of targets of DEGs revealed that, compared to mock-infected cells, CVB3-infected cells were differentially enriched in proteins associated with the inflammatory response and immune response (Figure 3A). Table 1 also shows that many of top 30 upregulated DEGs are associated with immune function. A heatmap representing 20 DEGs involved in immune function includes multiple antiviral response genes, such as *OAS1*, *OAS2*, *IFNB1*, *Mx1*, *Mx2*, and *CXCL10* (Figure 3B). Although expressions of immune-related genes were unchanged or downregulated at 4 hpi, gene expression of anti-viral response genes were highly upregulated at 24 hpi and continued to be increased even at later time points, including 48 and 72 hpi. Interestingly, genes involved in inflammation, such as *IL-6*, were also found to be among the highly regulated DEGs. Meanwhile, *IFNG1* gene expression was found to be significantly downregulated upon CVB3 infection (FC = 0.154 at all timepoints).

Interferon stimulated genes (ISGs) are anti-viral factors induced by IFNs in response to viral infection. DEGs identified in the previous step were imported into Cytoscape (version 3.3.0) for the identification of ISG-protein network analysis (Figure 3C). The nodes represent DEGs of 48 hpi samples, whose expression levels are depicted with colors corresponding to the fold change values. *OAS2* demonstrated the highest expression level in the network and many ISGs are found to interact with each other. In addition, a KEGG pathway enrichment analysis was performed to determine which functional pathways correlated with the gene expression changes observed and identify functional groups that are significantly enriched in response to CVB3 infection. As shown in Figure 3D, CVB3-induced DEGs in hNPCs were associated with retinoic acid-inducible gene I (RIG-I) signaling pathways, specifically upregulating *ISG15*, *RIG-I*, *LGP2*, *MDA5*, *IFN-γ*, *IL-8*, and *IP-10*.

### 3.3. Confirmation of Quant-Seq Data Using Quantitative RT-PCR Assay

**Figure 4:**
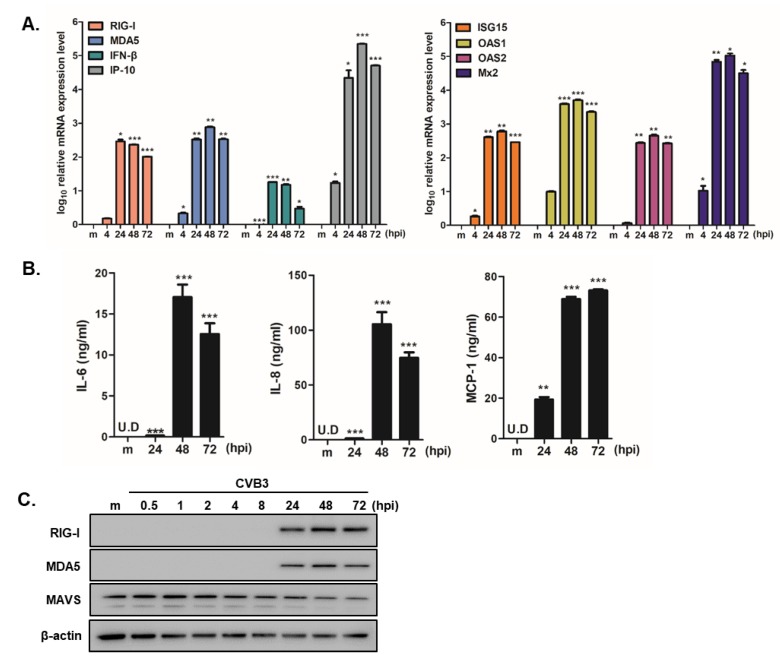
Interferon signaling and inflammatory responses are induced in CVB3-infected hNPCs. (**A**) Quantitative RT-PCR analysis was performed to measure *RIG-I*, *MDA5*, *IFN-β*, *IP-10*, *ISG-15*, *OAS1*, *OAS2*, and *Mx2* transcript levels following CVB3 infection (MOI 5) in hNPCs. * *p* < 0.05; ** *p* < 0.01; *** *p* < 0.001, compared with mock-infected cells. (**B**) The concentration of secreted inflammatory cytokines/chemokines (IL-6, IL-8, MCP-1) in the cultured media was determined by ELISA. * *p* < 0.05; ** *p* < 0.01; *** *p* < 0.001 versus mock-infected control cells (**C**) The protein levels of RIG-I, MDA5, MAVS, and β-actin were measured by western blotting.

To validate the QuantSeq results, we used a quantitative RT-PCR assay to analyze the expression levels of the selected upregulated genes. In accordance with the QuantSeq data, CVB3 infection in hNPCs exhibited a statistically significant increase in mRNA expression levels of *DDX58 (RIG-I)*, *IFIH1 (MDA5)*, *IFN-β*, *IP-10*, *ISG-15*, *OAS1*, *OAS2*, and *Mx2* compared to mock-control cells at 24, 48, and 72 hpi (Figure 4A). We also measured IL-6, IL-8, and MCP-1 secretion levels in the supernatants of CVB3-infected hNPCs by ELISA. Consistent with QuantSeq results, higher levels of cytokine/chemokine were released in a time-dependent manner (Figure 4B). Western blot also revealed enhanced RIG-I and MDA5 expressions following CVB3 infection in hNPCs, however the expression level of MAVS was downregulated upon CVB3 infection, as previously reported [25] (Figure 4C).

### 3.4. Type II IFN-Induced SOCS Upregulation Enhances CVB3 vRNA Expression

**Figure 5:**
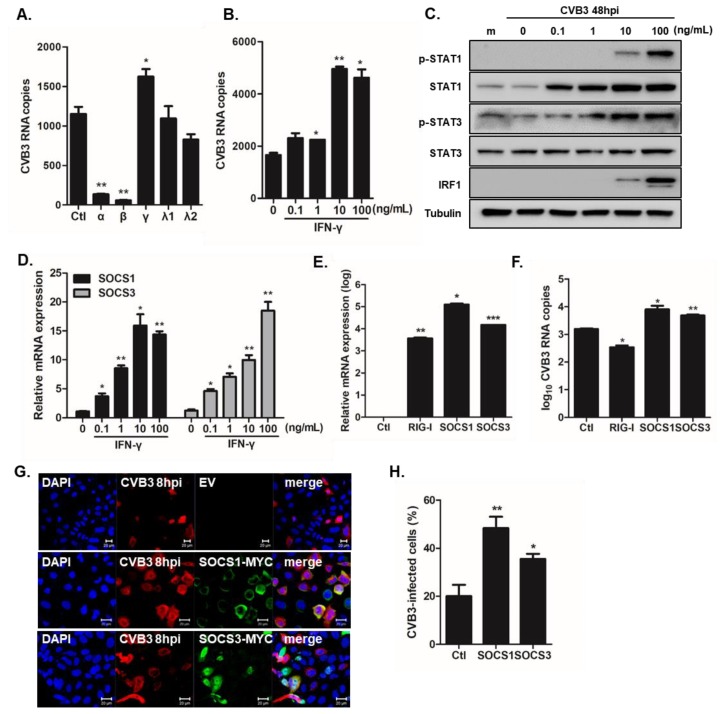
Type II interferon (IFN) signaling results in enhanced viral replication via upregulation of suppressor of cytokine signaling (SOCS)1 and SOCS3. (**A**) Recombinant human IFN-α (10 ng/mL), IFN-β (10 ng/mL), IFN-γ (10 ng/mL), IFN-λ1 (100 ng/mL), and IFN-λ2 (100 ng/mL) were added to hNPCs. The next day, cells were infected with CVB3 (MOI 5) and viral RNA expression was measured by RT-PCR. Data are shown as means ± SEM of three independent experiments. (**B**–**D**) hNPCs were treated with various doses of IFN-γ overnight, followed by CVB3 infection (MOI 5). (**B**) CVB3 copy number was determined (**C**) The protein levels of IFN-γ downstream signaling pathways, such as pSTAT1/STAT1, pSTAT3/STAT3, and interferon regulatory factor 1 (IRF1) were measured by western blotting. (**D**) RT-PCR was performed to measure relative *SOCS1* and *SOCS3* mRNA levels. * *p* < 0.05; ** *p* < 0.01; *** *p* < 0.001 versus mock-infected control cells. (**E**,**F**) HeLa cells were transfected with empty vector (EV), SOCS1-MYC tag, or SOCS3-MYC tag encoding plasmids for 24 h. The next day, cells were infected with CVB3 (MOI 1) for 8 hpi. Transfection efficiency was confirmed by measuring each gene expression level. Changes in the transcriptional expression of CVB3 vRNA were measured using RT-PCR. Transcript expression levels were calculated in relation to the expression level of β-actin and expressed as a fold-change in comparison to the expression level in EV-transfected control cells. * *p* < 0.05 versus mock-infected EV-transfected cells. (**G**,**H**) HeLa cells were transfected with EV, MYC-SOCS1, or MYC-SOCS3 encoding plasmids for 24 h and infected with CVB3 for 8 h. Cells were immunostained with anti-Coxsackievirus B3 antibody and anti-MYC antibody to detect SOCS proteins. CVB3 is indicated in red and cell nuclei are stained blue. The images are representative of three independent experiments. Scale bar represents 20 μm. CVB3-infected cells were counted and presented as % in the graph.

Given that IFN-β gene was among the most upregulated DEGs, while IFN-γ gene was significantly downregulated in QuantSeq analysis, we investigated the effect of differential IFN signaling and function in CVB3-infected hNPCs. Recombinant human IFNs (IFN-α, IFN-β, IFN-γ, IFN-λ1, and IFN-λ2) were added to the hNPCs. Cells were infected with CVB3 at an MOI of 5 for 8 h and Quantitative RT-PCR assay was performed to determine CVB3 RNA copy number. Figure 5A shows that type I IFN (IFN-α, IFN-β) treatment led to impaired CVB3 replication, while type II IFN treatment led to the opposite effect, causing enhanced viral replication in hNPCs. In addition, IFN-γ treatment exhibited a dose-dependent effect (Figure 5B), while IFN-γ treatment resulted in the time-dependent phosphorylation of Signal transducer and activator of transcription 1 (STAT1) and STAT3 (Figure 5C). Furthermore, specific upregulation of interferon stimulatory factor 1 following IFN-γ treatment was also observed, suggesting that IFN-γ signaling is functional. To determine the possible mechanisms by which IFN-γ signaling leads to enhanced viral replication, we next measured the expression levels of suppressor of cytokine signaling 1/3 (SOCS1/3) upon CVB3 infection in hNPCs. Dose-dependent increase of SOCS1 and SOCS3 gene expression levels following CVB3 infection occurred in hNPCs (Figure 5D). Next, we examined whether SOCS regulates CVB3 replication. Quantitative RT-PCR assay was utilized to quantify the mRNA fold-induction levels of SOCS1, SOCS3, and RIG-I. After confirming the overexpression levels of SOCS1 and SOCS3 (Figure 5E), we determined the transcript level of CVB3 RNA copy number in response to the overexpression. Overexpression of SOCS1 or SOCS3 resulted in a significant increase of CVB3 RNA replication, whereas overexpression of RIG-I resulted in reduced CVB3 RNA copy number (Figure 5F). Additionally, increased CVB3 immunostaining in SOCS1 or SOCS3 overexpressing cells highlight the importance of SOCS expression during a productive viral replication (Figure 5G,H).

## 4. Discussion

*Enteroviruses*, including Coxsackieviruses (CVs), surprisingly exhibit a tropism for the central nervous system (CNS) and are commonly associated with viral meningitis and encephalitis [3]. The adult CNS contains NPCs with self-renewable, multipotent characteristics for neurogenesis and plasticity [29]. hNPCs give rise to glial and neuronal cell types that produce a diverse array of secreted mediators and receptors, all of which are relevant for the maintenance of the neurogenic niche [30]. Neurotropic viruses can disrupt the survival, proliferation, and maturation of NPCs, and ultimately impair neurogenesis. In particular, the effect of neurotropic virus infection on hNPCs has been well demonstrated through extensive research. Zika virus, in particular, directly infects human neural progenitor cells and results in transcriptional dysregulation and attenuated cell growth, in addition to cytotoxic effects, leading to the abrogation of neurogenesis [31,32,33,34].

Previous studies using mice models have demonstrated the ability of CVB3 to infect proliferating NPCs located in the neonatal subventicular zone and persist in the adult murine CNS [5,7]. Furthermore, the persistence of CVB3 within the murine neurogenic region can lead to the infection of neural stem cells, causing cell death, decrease in brain size, and eventually developmental defects [8]. Since CVB3-induced spontaneous abortions, fetal myocarditis, and neurodevelopmental delays in the newborn can result in fatal outcomes [35], the characterization of CVB3 pathogenesis in the hNPC model is essential.

In this study, we examined comprehensive gene expression profiling of CVB3-infected hNPCs. Our data reveal that hNPCs are susceptible to CVB3 infection, while no minimal cytopathic effect was observed upon CVB3 infection. QuantSeq data indicate that the most highly regulated DEGs were involved in biological pathways associated with RIG-I antiviral innate immunity and inflammatory responses. In contrast to the upregulation of type I IFNs, the *IFN-γ* gene was downregulated upon CVB3 infection. Furthermore, the treatment of recombinant IFN-γ protein in hNPCs led to the upregulation of SOCS proteins, thus facilitating viral replication.

Cytoplasmic helicase receptors, including retinoic acid-inducible gene I (RIG-I) and melanoma differentiation-associated gene 5 (MDA5), are important mediators of intracellular viral nucleic acid sensing. Each consists of a C-terminal DEXD/H-box RNA-helicase domain and an N-terminal caspase recruitment domain and can induce IFN gene transcription in response to viral RNA. In mice, MDA5, but not RIG-I, seems to play as a predominant receptor for immune protection against CVB3 infection [11]. Meanwhile, our QuantSeq data highlight alterations in expression patterns of host genes during CVB3 infection in hNPCs. Many DEGs involved in both RIG-I and MDA5-mediated antiviral signaling were detected to be upregulated, in addition to DEGs involved in inflammatory pathways. Likewise, an RT-PCR analysis was performed to validate the QuantSeq data, and the transcript levels of interferon stimulated genes, such as of *DDX58 (RIG-I)*, *IFIH1 (MDA5)*, *IFN-β*, *IP-10*, *ISG-15*, *OAS1*, *OAS2*, and *Mx2* were all upregulated in response to CVB3 infection. Several molecules that increase the inflammatory response in the brain, such as CXCL10 and IL-6, were upregulated in CVB3-infected hNPCs. Interestingly, the significant suppression of *IFN-γ* gene in CVB3-infected hNPCs proved to be an unexpected finding. IFN-γ, a pleiotropic cytokine, has a central role in regulating NSCs proliferation and quiescence [36,37]. Li et al. previously demonstrated that K3 and K5 proteins of Kaposi’s sarcoma-associated herpesvirus specifically target gamma interferon receptor 1 (IFN-γR1) and induce its ubiquitination, endocytosis, and degradation to downregulate IFN-γR1 surface expression, and, thereby, inhibit IFN-γ action [38]. It is possible that CVB3 also targets IFN-γR1 to downregulate IFN-γ activity in hNPCs.

The SOCS family of proteins is involved in the regulation of both innate and adaptive immunity, including negative regulation of JAK/STAT pathways, dendritic cell activation, T cell differentiation and function [39,40]. Among the eight members of the SOCS family, SOCS1 and SOCS3 have an amino-terminal kinase inhibitory region that inhibits JAK tyrosine kinase activity and a carboxy-terminal SOCS box that recruits the ubiquitin transferase complex [41,42]. This multifaceted structure enables SOCS proteins to possess a wide range of biological functions [43]. Recently, SOCS proteins have gained attention as frequent targets of viral exploitation of the host immune response. Multiple viruses promote their survival by inducing SOCS1 and/or SOCS3 protein expression following infection [44,45,46,47,48,49,50,51,52]. Here, for the first time, we show that IFN-γ activation followed by CVB3 infection can result in the upregulation of SOCS expression in hNPCs and SOCS overexpression facilitates CVB3 replication. Nonetheless, further studies are required to characterize the primary roles and immunomodulatory mechanisms of SOCS proteins during CVB3 infection in particular.

Of note, Huber and colleagues have shown that several wild type CVB3 variants exist and they were different in their capacity to cause myocarditis [14,53,54]. In our study, we used the CVB3 H3 Woodruff variant, which presented high susceptibility for myocardial cells in vitro and lacked tropism for heart in vivo [9]. Interestingly, CVB3 may recombine with other Enterovirus B viruses, like Echo-6, Echo-16, Echo-30, Echo-25, and CVB5 [55]. Thus, in a clinical setting, although the virus may be identified as CVB3, based on the VP1 sequence, it is still possible that the recombinant sequence of a patient may have different properties and the cellular response may be different. Therefore, it will be important to further characterize the pathogenic effect of these recombinant strains in cellular and animal models.

In conclusion, our study provides evidence for the involvement of antiviral and inflammatory pathways during CVB3 infection in hNPCs. Additional in vitro and in vivo studies are necessary for further validation of the role of different IFNs and SOCS proteins, as well as their contribution to the outcome of CVB3-induced neurological diseases. Identifying pathways or markers of viral neurological diseases will shed light on the development of novel therapeutic targets or strategies to prevent or treat enterovirus infections, and ultimately contribute to the alleviation of morbidity and mortality caused by these infections.

## Figures and Tables

**Table 1:** Top 30 up-regulated differentially expressed genes in CVB3-infected hNPCs.

CVB3 4 hpi		CVB3 24 hpi		CVB3 48 hpi		CVB3 72 hpi	
Gene Symbol	Fold Change	Gene Symbol	Fold Change	Gene Symbol	Fold Change	Gene Symbol	Fold Change
ALB	199.710911	OAS2	3590.752076	CXCL10	2788.729472	OAS2	2144.693059
FGL1	151.232253	OASL	3157.978963	OAS2	2585.682507	C3	1370.949319
RNF128	81.41198341	OAS1	1992.55583	OASL	2106.305157	OAS1	1273.963344
SERPINF2	30.54163219	TNFSF10	1361.117621	OAS1	1849.216367	XAF1	1113.846352
SLC16A1-AS1	25.72680704	MX1	1328.717762	RTP4	1343.713889	RTP4	1104.350773
ELAVL4	21.32235069	MX2	1167.395062	CFB	1288.595659	CFB	993.7473701
PDZRN4	21.17101413	RSAD2	1093.135109	UBD	1259.348916	CXCL10	962.3589151
ADIPOQ	19.45340008	IFNL1	1048.127664	IL6	1239.857521	UBD	912.9052931
SNORD116-19	19.41086946	CXCL10	1028.293071	CCL5	1204.757213	OASL	911.0220382
NEK10	18.41598152	XAF1	1027.553381	C3	1190.750612	IFI27	900.0510031
QRICH2	17.49609503	IFIT2	788.4576455	XAF1	1178.838354	IL6	531.8343858
LST1	17.41151256	RTP4	768.0683629	TNFAIP6	1123.497903	MX2	529.4197535
GRB7	16.20838037	IL4I1	688.7841437	CSF3	1104.636105	IL4I1	513.8757802
FAM223A	15.72756064	TMEM229B	678.5905614	IFI27	1079.775496	CCL5	440.3891312
CLEC4A	15.68990948	IFIT1	623.389733	MMP13	973.8776634	TNFSF10	427.3208197
KCNK15	14.9799394	CMPK2	583.097432	CCL20	880.6629597	CMPK2	411.5838874
FGB	14.16735269	IFI27	565.226547	TNFSF10	834.3706653	GJD3	374.5633629
RIMS4	14.10446292	DHX58	564.786182	MX2	820.6524514	MX1	366.8709422
VSX1	13.92185119	BATF2	557.3910666	IL4I1	719.9424761	TLR2	330.9557696
LRMP	13.91077957	HERC5	518.9686208	C15orf48	644.935481	TNFAIP6	323.5636471
NRN1	13.89623352	IFNB1	456.2776531	DHX58	604.6042633	DHX58	317.7420517
PDHA2	13.87089374	ISG15	438.5791237	CD38	582.5380113	HCP5	307.3736554
NOXA1	13.77357575	IFIH1	428.6527181	APOL3	573.7072597	MMP13	302.4400874
PLCG2	13.62274943	USP30-AS1	409.4871834	RSAD2	558.735795	RSAD2	302.4042005
C7orf66	13.21422234	ISG20	354.2738372	CXCL11	548.2025904	TMEM229B	296.7585139
PROC	13.11771717	LGALS9	354.1803021	CMPK2	534.5673803	CH25H	288.5077209
LOC100289580	13.01231576	HRASLS2	335.0680854	ISG20	497.0178895	HLA-F	287.6274767
GRIA3	13.00956168	HLA-F	325.6220895	MX1	487.8623426	BATF2	278.754509
DCDC1	12.92863814	TNFSF13B	318.3349541	HLA-F	422.0001454	ISG20	255.3614733
NOXRED1	12.91702462	APOL6	303.6140085	RARRES1	404.1442974	CD38	249.3775165
